# Mangosteen (*Garcinia mangostana*) flesh supplementation attenuates biochemical and morphological changes in the liver and kidney of high fat diet-induced obese rats

**DOI:** 10.1186/s12906-019-2764-5

**Published:** 2019-12-02

**Authors:** Noratirah Shazlin Muhamad Adyab, Asmah Rahmat, Noor Atiqah Aizan Abdul Kadir, Hawa Jaafar, Radhiah Shukri, Nurul Shazini Ramli

**Affiliations:** 1 Faculty of Medicine and Health Sciences, Universiti Putra Malaysia, 43400 UPM, Serdang, Malaysia; 2 Faculty of Science, Technology and Human Development, Universiti Tun Hussein Onn, Malaysia (UTHM), 86400 Parit Raja, Batu Pahat, Malaysia; 3 Faculty of Agriculture, Universiti Putra Malaysia, 43400 UPM, Serdang, Selangor Darul Ehsan Malaysia; 4 Faculty of Food Science and Technology, Universiti Putra Malaysia, 43400 UPM, Serdang, Malaysia

**Keywords:** *Garcinia mangostana*, Flesh - antioxidant, Inflammation, Xanthones, Liver, Kidney

## Abstract

**Background:**

Mangosteen is a native fruit from Southeast Asia. It is rich in phenolic compounds such as xanthones, anthocyanins and phenolic acids. Mangosteen pericarp extract showed inhibitory activity towards pancreatic lipase and may have potential use for obesity treatment. However, there is limited study on the beneficial effects of mangosteen flesh against obesity. This study aimed to investigate the effects of *Garcinia mangostana* flesh (GMF) on biochemical and morphological changes in the liver and kidney of high-fat diet-induced obese rats.

**Methods:**

Forty healthy Sprague-Dawley rats were randomised into five groups (*n* = 8) with four groups were fed with high-fat diet (HFD) for 10 weeks and a control group was fed with rat chow diet. Supplementation with GMF in obese rats was continued for 7 weeks starting from week 10th after the initiation of HFD at different doses (200 mg/kg, 400 mg/kg and 600 mg/kg). The positive and negative control rats were given distilled water via oral gavage. Plasma lipid profile, antioxidant enzymes and pro-inflammatory markers were determined using commercial kits. Liver and kidney structure were defined by histology.

**Results:**

The rats fed with HFD for 10 weeks increased plasma LDL-cholesterol, reduced plasma glutathione peroxidase level and had significantly higher body weight compared to normal control rats (*p* < 0.05). Obese rats also showed elevated level of TNF-α and IL-6 after 17 weeks of HFD. Supplementation with GMF for 7 weeks in obese rats reduced their body weight, improved lipid profile, increased total antioxidant capacity and glutathione peroxidase level and lowered plasma pro-inflammatory markers (TNF-α and IL-6) (*p < 0.05*). In addition, GMF supplementation attenuated the abnormalities of the liver and kidney tissue caused by high fat diet.

**Conclusion:**

Taken together, the findings suggest that supplementation of *Garcinia mangostana* flesh may help in reducing body weight and has the potential to ameliorate the biochemical changes of the high fat diet-induced obesity in rats. Further studies on pharmacodynamic and pharmacokinetic are required before the results are translated to human.

## Background

Obesity appeared to be a leading health challenge over the past century in both developed and developing countries. According to World Health Organization [1], the prevalence of worldwide obesity tripled between 1975 and 2016 with the current statistics showed 13% of adults aged 18 and above were obese and 39% were overweight. Obesity is characterized by excess accumulation of fat that lead to increase body weight [1]. It is associated with adverse health problems with fatal complications including insulin resistance, enhanced inflammatory marker expression, obstructive sleep apnoea syndrome and increased oxidative stress. Consequently, the number of people with hypertension, diabetes mellitus and cardiovascular diseases are proportionally increase with the growing prevalence of obesity [2]. The aetiology of obesity is very complex involving genetic, environmental and psychosocial factors acting through the physiological mediators of energy intake and expenditure [3]. Data from several studies suggest that increase consumption of high-calorie foods coupled with reduce intake of fruits and vegetables favour a positive energy balance which set the foundation for obesity epidemic [4–7]. On the contrary, diet rich in vegetables and fruits can decrease the risk for chronic diseases through the beneficial combinations of fibers, micronutrients, and phytochemicals [8, 9].

The economic burden of obesity including the tangible and intangible cost is projected to increase every year [1]. Therefore, the development of novel natural therapy that is safe and effective is very critical in order to alleviate the global public health crisis of obesity and its related co-morbidities. To date, only few prescription drugs available and approved by Food and Drug Administration (FDA) for obesity treatments [10]. However, the use of anti-obesity drugs produces only small and inconsistent weight loss with severe adverse effects including abdominal pain, faecal urgency and incontinence [11]. Alternatively, dietary intervention including the use of plant based extract could produce a consistent decrease in obesity and its co-morbid diseases with less side effects.

*Garcinia mangostana* or known as mangosteen is a dark purple fruit with slightly acidic and sweet flavour flesh. The fruit is commonly found in the Asian region like Malaysia, India and Thailand. The fruit pericarps have been traditionally used to treat various medical conditions such as abdominal pain, wounds and skin infection since ancient time [12]. Numerous bioactive compounds have been isolated from different parts of mangosteen [13]. Among them are xanthones derivatives which are the major constituent in the pericarp. Extensive research has been carried out on the mangosteen pericarp, and demonstrated a wide range of activities including antifungal, antioxidant, antiobesity and antidiabetic [14].

The ethanolic extract of *G. mangostana* has been reported to exhibit potent hypoglycemic effect in streptozotocin-induced diabetic rats [15]. It is hypothesized that the positive effects were due to inhibition of α-glucosidase and α-amylase activity, which are the enzymes that responsible for carbohydrate digestion [16]. In an in-vitro study, the major component of *G. mangostana* extract, α-mangostin and γ-mangostin showed inhibition toward the production of pro-inflammatory markers in RAW.264.7 macrophage cells [17]. Moreover, Bumrungpert et al. [18] demonstrated that xanthones from mangoesteen ameliorated inflammation in primary human adipocytes by decreasing the expression of inflammatory mediators. Further clinical research exploring the effect of mangosteen juice blend supplementation in 40 subjects for 8 weeks found a reduction in body mass index (BMI) and c-reactive protein (CRP) levels [19]. Inflammation has been implicated in various chronic diseases including obesity. Despite of numerous reports on anti-inflammatory activities of mangosteen, to the best of our knowledge, no single study exists that examine the effect of mangosteen flesh on obesity. Hence, the present study was carried out to investigate the potential of *Garcinia mangostana* flesh (GMF) in attenuating the metabolic and structural changes in high fat diet-induced obese rats. In this study, Sprague Dawley rats were used because they produced neural and microvascular abnormalities which are a more-human like model after induction with high fat diet [20]. Besides, diet-induced model was found to be the closest model that have comparable aetiology of obesity as well as the pathophysiology changes that occur in obese human [21, 22].

## Methods

### Plant identification

The formal identification of *G. mangostana* was conducted by a botanist, Dr. Mohd Firdaus Ismail, from Biodiversity Unit, Institute of Bioscience, Universiti Putra Malaysia against the voucher specimen. The voucher no. is SK 3283/18. The sample was also deposited in the Herbarium of the Institute of Bioscience, Universiti Putra Malaysia.

### Preparation of *G. mangostana* flesh

Mature *G. mangostana* was characterized by dark purple rinds and pleasant smell [23]. The fruits were bought from the local market, Selangor, Malaysia. The fruits were rinsed with water and the pericarps were removed. The fruit’s flesh was stored at − 80 °C and freeze dried to remove the moisture content. Dried *G. mangostana* flesh (GMF) was ground into fine powder and kept in air tight container at 4 °C. During the supplementation period, the GMF was freshly prepared daily by diluting with distilled water at three concentrations (200 mg/kg, 400 mg/kg and 600 mg/kg) before given to the experimental animals.

### Experimental animals

The experimental procedures were conducted according to ethical norms approved by Animal Care and Use Committee (ACUC) of the Universiti Putra Malaysia (UPM/IACUC/AUP-R014/2014). Forty male Sprague Dawley rats (aged 8–10 weeks, weight 250 ± 5 g) were purchased from A Sapphire Enterprise (Malaysia). All rats were healthy as reported by the supplier. The rats were individually housed in a standard cage at the Universiti Putra Malaysia animal house in a temperature controlled at 28 ± 2 °C with 12 h light and dark cycle. Some of the male rats were aggressive. Therefore, they were individually housed but in the same room to ensure they maintain olfactory, auditory and visual contact with other rats, and thus they were not totally isolated. Acclimatization was conducted for one week by giving ad libitum water and rat chow pellet.

### Induction of obesity

High-fat diet (HFD) was adapted based on the diet composition previously reported by Levin and Dunn-Meynell [13]. The HFD consisted of 68% normal rat chow pellet, 20% instant milk powder (Dutch Lady), 6% ghee (Crispo) and 6% coin oil (Krystal). The mixture were homogenized and baked overnight at 65 °C. The HFD provides 414.0 kcal / 100 g of energy with 43% carbohydrate, 17% protein and 40% fat. Meanwhile, rat chow pellet contains 306.2 kcal/100 g of energy with 75% carbohydrate, 22% protein and 3% fat.

### Animal grouping

The rats were randomly divided into 5 groups according to their diet: rat chow pellet (normal control, NC; *n* = 8), HFD (obese control, OC; *n* = 8), HFD + 200 mg /kg GMF (GMF 200; *n* = 8), HFD + 400 mg /kg GMF (GMF 400; *n* = 8) and HFD + 600 mg /kg GMF (GMF 600; *n* = 8). The respective dosages were based on the study by Adiputro et al. [23]. The rats that received HFD for 10 weeks had significantly higher body weight and body mass index (BMI) and were classified as obese [14]. GMF supplementation was administered for 7 weeks in obese rats starting from week 10th after the initiation of HFD. Meanwhile, NC and OC groups received placebo, an inactive substance (distilled water) through force feeding (oral gavage) for 7 weeks starting from week 10th after the initiation of normal diet and HFD respectively as the total volumes of the treatment is less than 5 ml/kg. All the measurements including body weight and other experiments were conducted in the light phase.

### Food intake, body weight and body mass index (BMI)

Daily food intake was taken to monitor the health status of the rats. The leftover food within 24 h was weighed using an electrical balance before deducted from the initial food weight. Body weight measurement was recorded weekly while the measurement of naso-anal length (nose to anus) was recorded at week 10 to determine their BMI. BMI was calculated by dividing body weight (g) with naso-anal length (cm^2^). Normal BMI for adult rats ranged between 0.45 ± 0.02 g/cm^2^ and 0.68 ± 0.05 g/cm^2^ [14].

### Plasma biochemistry analysis

Blood collection was performed at week 10 and week 17 of dietary intervention and kept into heparinized tubes. Blood was collected from the lateral saphenous vein at week 10 by making a puncture using a fine needle, and the blood flow was stopped by pressing gently on the puncture site. At week 17, terminal anaesthesia was induced via intraperitoneal injection of pentobarbitone sodium (Lethabarb, 100 mg/kg), and blood was drawn via cardiac puncture. Heparin (Sigma-Aldrich Australia) was administered (200 IU) through the right femoral vein. Plasma lipid profile (total cholesterol, triglyceride, high density lipoprotein (HDL)-cholesterol and low density lipoprotein (LDL)-cholesterol) analysis was done by using diagnostic reagent test kit (Roche, Germany). Plasma glucose was determined using glucose oxidase assay kit (Sigma-Aldrich, USA) using Hitachi Automatic Analyzer (Tokyo, Japan). Plasma total antioxidant capacity (TAC), superoxide dismutase (SOD) and glutathione peroxidase (GPx) activity were determined using kits and controls based on manufacturer’s standard protocols (RANDOX Laboratory Ltd., USA) using a clinical chemistry analyzer machine (Vitalab Selecktra E, Germany). Plasma inflammatory markers namely alpha-tumor necrosis factor (α-TNF) and inteleukin-6 (IL-6) were analyzed using Platinum ELISA kit (eBioscience, Vienna, Austria) according to manufacturer’s instructions.

### Histopathological examination

At the end of study, the rats were euthanized by exsanguination. The liver and kidney were washed in ice-cold 0.95 NaCl solutions and kept in specimen container with 10% buffered formalin. Once fixed, the tissues were embedded in paraffin and stained hematoxylin and eosin (H&E). Lastly, slides were prepared and were observed under 20X magnifications using light microscopy.

### Statistical analysis

All data were analysed using Statistical Package for Social Science software (SPSS version 22). Data were expressed as mean ± SEM. A total of 5 groups were analysed using one-way analysis of variance (ANOVA). Each group consists of eight rats and the experimental unit was an individual rats. All group data were tested for variance using Bartlett’s test. Variables that were not normally distributed were transformed (using log 10 function) prior to statistical analysis. When interaction and/or the main effects were significant, means were compared using Newman-Keuls multiple-comparison post hoc test. Where transformations did not result in normality or constant variance, a Kruskal-Wallis nonparametric test was performed. The data was considered statistically significant at *p < 0.05*.

## Results

### Dietary intake and body weight

The effects of GMF supplementation on dietary intake, body weight and BMI in high-fat diet-induced obese rats was shown in Table 1. After 10 weeks of HFD, obese rats showed lower food intake compared to normal control rats (*p* < 0.05) (Table 1). However, energy intake was higher in obese groups and this was associated with higher body weight and BMI compared to normal control group (*p* < 0.05) (Table 1).

**Table 1:** The effect of GMF supplementation on dietary intake, body weight and BMI

Variables			Groups		
	NC	OC	GMF 200	GMF 400	GMF 600
Food intake (g/day)					
Week 0–10	23.83 ± 0.41^a^	20.55 ± 0.53^b^	18.57 ± 0.72^c^	18.76 ± 0.30^c^	17.98 ± 0.22^c^
Week 10–17	24.14 ± 0.66 ^b^	24.93 ± 0.50 ^a^	21.76 ± 1.20^c^	21.00 ± 0.40^c^	22.81 ± 0.45^bc^
Energy intake (kcal/day)					
Week 0–10	72.97 ± 1.25^b^	85.07 ± 2.20^a^	76.89 ± 3.00^b^	77.67 ± 1.24^b^	74.42 ± 0.92^b^
Week 10–17	73.92 ± 2.01^c^	103.21 ± 0.20^a^	90.10 ± 5.00^b^	86.94 ± 1.64^b^	94.43 ± 1.84^b^
Body weight (g)					
Week 0	233.17 ± 6.49^a^	237.17 ± 5.97^a^	232.33 ± 6.14^a^	233.33 ± 6.16^a^	235.50 ± 4.54^a^
Week 10	397.17 ± 17.24^b^	476.50 ± 11.50^a^	468.67 ± 11.42^a^	477.3311.20^a^	476.83 ± 9.26^a^
Week 17	453.17 ± 19.67^b^	565.83 ± 11.98^a^	484.50 ± 13.57^b^	488.50 ± 12.02^b^	503.17 ± 9.15^b^
Weight gain (10th -17th week)	56.00 ± 2.43^b^	81.33 ± 0.48^a^	15.83 ± 2.15^d^	11.17 ± 0.82^d^	26.34 ± 0.11^c^
BMI (g/cm^2^)					
Week 10	0.56 ± 0.02^b^	0.70 ± 0.01^a^	0.69 ± 0.02^a^	0.69 ± 0.004^a^	0.70 ± 0.05^a^
Week 17	0.62 ± 0.03^b^	0.71 ± 0.03^a^	0.67 ± 0.02^b^	0.67 ± 0.03^b^	0.68 ± 0.06^b^

Each value represent mean ± SEM (*n* = 8). Means with different superscript letters within a row differ significantly (*p* < 0.05). NC, normal control; OC, obese control; GMF 200, 200 mg *G. mangostana* flesh / kg body weight; GMF 400, 400 *G. mangostana* flesh / kg body weight; GMF 600, 600 *G. mangostana* flesh / kg body weight; Energy intake is calculated based on macronutrient composition (carbohydrate, 4 kcal/g; fat, 9 kcal/g; protein 4 kcal/g)

Supplementation of GMF in obese rats for 7 weeks reduced food intake compared with NC and OC groups (Table 1). The energy intake was lower in GMF supplemented rats at all doses (GMF 200, GMF 400 and GMF 600) compared to OC group while NC group had the lowest energy intake (*p* < 0.05) (Table 1). All GMF supplementation groups had significantly lower body weight and BMI than OC group (*p* < 0.05) (Table 1).

### Plasma glucose and lipid profile

Rats fed with HFD at both 10 and 17 week had higher plasma glucose and LDL-cholesterol level compared with the NC group (*p* < 0.05) (Table 2). GMF supplementation at all doses for 7 weeks did not affect plasma glucose concentration (*p* < 0.05) (Table 2). In contrast, GMF supplementation at 400 mg/kg and 600 mg/kg reduced plasma LDL-cholesterol (*p* < 0.05) (Table 2). Obese rats also had greater plasma total cholesterol level at 10 week compared with NC rats although the difference was not significant (Table 2). Similarly, there were no differences in plasma triglyceride and HDL-cholesterol level for all the groups at week 10 (Table 2). Nevertheless, OC rats showed greater plasma total cholesterol and triglyceride concentration at week 17 compared to NC rats (*p* < 0.05) (Table 2). All GMF supplemented groups had significantly lower total cholesterol level than OC group after 7 weeks of GMF supplementation (*p* < 0.05) (Table 2). Besides, GMF 400 group had significantly lower triglyceride level compared to OC group (*p* < 0.05) (Table 2).

**Table 2:** The effect of GMF supplementation on plasma glucose and lipid profile

Variables			Groups		
	NC	OC	GMF 200	GMF 400	GMF 600
Glucose (mmol/L)					
Week 10	6.13 ± 024^b^	7.28 ± 0.26^a^	7.82 ± 0.39^a^	7.33 ± 0.22^a^	7.32 ± 0.30^a^
Week 17	6.53 ± 0.36^b^	7.73 ± 0.24^a^	7.45 ± 0.17^a^	7.67 ± 0.26^a^	7.75 ± 0.09^a^
Total Cholesterol (mmol/L)					
Week 10	1.35 ± 0.13 ^b^	1.61 ± 0.09^ab^	1.85 ± 0.12 ^a^	1.50 ± 0.21^ab^	1.74 ± 0.09^ab^
Week 17	1.19 ± 0.13^b^	2.20 ± 0.23^a^	1.43 ± 0.21^b^	1.11 ± 0.13^b^	1.30 ± 0.10^b^
Triglyceride (mmol/L)					
Week 10	0.71 ± 0.17 ^a^	0.44 ± 0.05 ^a^	0.62 ± 0.08 ^a^	0.61 ± 0.07 ^a^	0.63 ± 0.09 ^a^
Week 17	0.53 ± 0.11^b^	0.83 ± 0.11^a^	0.63 ± 0.11^ab^	0.46 ± 0.06^b^	0.59 ± 0.04^ab^
HDL-cholesterol (mmol/L)					
Week 10	1.12 ± 0.11^b^	1.21 ± 0.05^b^	1.55 ± 0.10^a^	1.19 ± 0.17^b^	1.41 ± 0.07^ab^
Week 17	0.94 ± 01.2^ab^	1.32 ± 0.21^a^	1.17 ± 0.19^ab^	0.79 ± 0.14^b^	1.06 ± 0.08^ab^
LDL-cholesterol (mmol/L)					
Week 10	0.05 ± 0.04^b^	0.20 ± 0.03^a^	0.33 ± 0.06^a^	0.21 ± 0.04^a^	0.21 ± 0.07^a^
Week 17	0.18 ± 0.05^b^	0.67 ± 0.37^a^	0.19 ± 0.04^ab^	0.12 ± 0.03^b^	0.12 ± 0.04^b^

Each value represent mean ± SEM (*n* = 8). Means with different superscript letters within a row differ significantly (*p* < 0.05). NC, normal control; OC, obese control; GMF 200, 200 mg *G. mangostana* flesh / kg body weight; GMF 400, 400 *G. mangostana* flesh / kg body weight; GMF 600, 600 *G. mangostana* flesh / kg body weight

### Antioxidant enzymes and total antioxidant capacity

HFD feeding for 10 and 17 weeks lowered GPx level compared to NC rats (*p* < 0.05) (Table 3). On the other hand, there was no difference in SOD level between NC and OC groups at week 10. At week 17, OC rats showed lower plasma SOD concentration compared to NC rats (*p* < 0.05) (Table 3). GMF supplementation increased plasma GPx, but not SOD, concentrations in all GMF groups. Moreover, GMF 400 and GMF 600 groups had the highest plasma GPx concentration among the groups (*p* < 0.05) (Table 3). There was no difference in plasma TAC level at week 10 in all groups (Table 3). At week 17, all GMF supplemented groups showed improved ability to scavenge free radicals as shown by higher TAC level compared to NC and OC groups respectively (*p* < 0.05) (Table 3).

**Table 3:** The effect of GMF supplementation on plasma antioxidant enzymes and total antioxidant status

			Groups		
Antioxidant enzymes	NC	OC	GMF 200	GMF 400	GMF 600
GPx (U/L)					
Week 10	1116.73 ± 79.59^a^	791.40 ± 97.10^b^	775.65 ± 135.11^b^	782.10 ± 114.14^b^	759.40 ± 123.56^b^
Week 17	1144.85 ± 42.19^b^	704.42 ± 100.20^c^	1403.20 ± 81.57^b^	1509.13 ± 80.35^a^	1735.28 ± 122.21^a^
SOD (U/ml)					
Week 10	7.62 ± 0.10^a^	7.53 ± 0.09^a^	7.50 ± 0.10^ab^	7.11 ± 0.13^b^	7.49 ± 0.08^a^
Week 17	7.22 ± 0.41^a^	5.96 ± 0.27^b^	5.98 ± 0.24^b^	6.08 ± 0.32^b^	6.11 ± 0.30^b^
TAC (mmol/L)					
Week 10	1.57 ± 0.02	1.45 ± 0.11	1.53 ± 0.08	1.53 ± 0.08	1.55 ± 0.11
Week 17	1.20 ± 0.04^b^	1.27 ± 0.07^b^	1.52 ± 0.07^a^	1.55 ± 0.07^a^	1.63 ± 0.08^a^

Each value represent mean ± SEM (*n* = 8). Means with different superscript letters within a row differ significantly (*p* < 0.05). NC, normal control; OC, obese control; GMF 200, 200 mg *G. mangostana* flesh / kg body weight; GMF 400, 400 *G. mangostana* flesh / kg body weight; GMF 600, 600 *G. mangostana* flesh / kg body weight; GPx, glutathione peroxidase; SOD, superoxide dismutase; TAC, total antioxidant capacity

### Inflammatory markers

Compared with NC rats, the OC group had elevated plasma TNF-α and IL-6 after 17 weeks of HFD (*p* < 0.05) (Table 4). GMF supplementation at all doses (GMF 200, GMF 400 and GMF 600) ameliorated these changes by reducing TNF-α and IL-6 concentration (*p < 0.05*) (Table 4). GMF supplemented rats showed the lowest plasma TNF-α concentration compared to NC and OC rats respectively (*p* < 0.05) (Table 4). Although GMF supplemented groups showed the trend of reducing plasma IL-6 with increasing GMF dose, the differences were not significant (*p* > 0.05) (Table 4).

**Table 4:** The effect of GMF supplementation on inflammatory markers

Pro-inflammatory markers			Groups		
	NC	OC	GMF 200	GMF 400	GMF 600
TNF-α (pg/ml)					
Week 17	74.00 ± 17.47^b^	128.58 ± 29.66^a^	41.24 ± 15.60^c^	46.43 ± 18.76^c^	77.62 ± 38.01^c^
IL-6 (pg/ml)					
Week 17	4907.67 ± 793.34^b^	12,093.33 ± 2740.55^a^	2922.67 ± 303.61^b^	2746.17 ± 781.68^b^	2431.00 ± 433.58^b^

Each value represent mean ± SEM (*n* = 8). Means with different superscript letters within a row differ significantly (*p* < 0.05). NC, normal control; OC, obese control; GMF 200, 200 mg *G. mangostana* flesh / kg body weight; GMF 400, 400 *G. mangostana* flesh / kg body weight; GMF 600, 600 *G. mangostana* flesh / kg body weight; TNF-α, tumor necrosis factor alpha; IL-6, interleukin 6

### Histopathological investigation

**Fig. 1:**
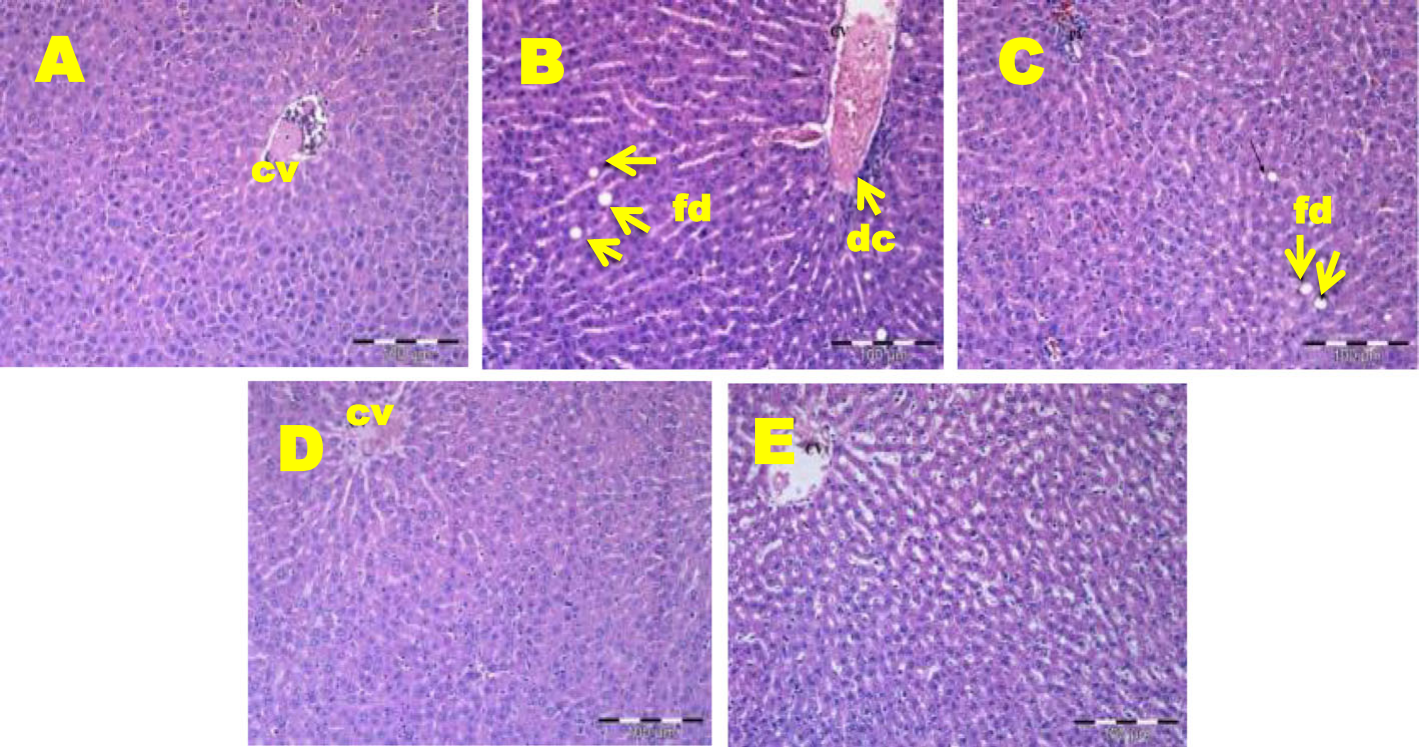
Histopathology of liver in NC, OC and GMF supplemented rats. Haematoxylin and eosin staining of liver tissues showing enlarged hepatocytes and fat droplets (marked as “fd”) (× 20), and dilated central vein (marked as “dc”) (× 20). CV, central vein; **a**, normal control (NC); **b**, obese control (OC); **c**, 200 mg *G. mangostana* flesh / kg body weight (GMF 200); **d**, 400 *G. mangostana* flesh / kg body weight (GMF 400); **e**, 600 *G. mangostana* flesh / kg body weight (GMF 600)

Histopathological examination of liver tissues showed enlarged hepatocytes and appearance of fat droplets in OC rats after 17 weeks of HFD feeding (Fig. 1b). In contrast, NC rats showed normal hepatocytes with small amount of fat and no inflammatory cells (Fig. 1a). The GMF 200 group had less fat cell accumulation (Fig. 1c) while GMF 400 and GMF 600 groups normalized hepatocytes irregularities of OC group (Fig. 1d and e).

**Fig. 2:**
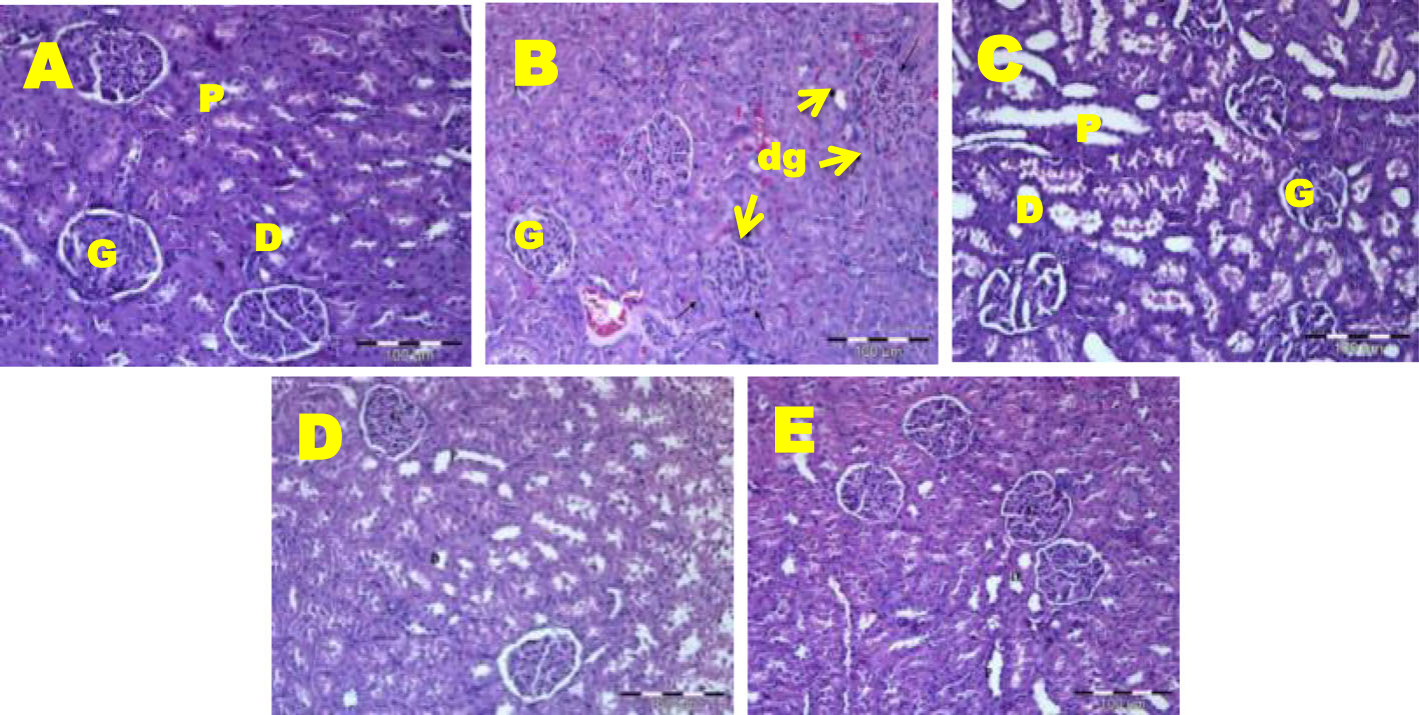
Histopathology of kidney in NC, OC and GMF supplemented rats. Haematoxylin and eosin staining of renal tissues showing dilated glomerulus (marked as “dg”) (× 20). G, Glomeruli with Bowman’s capsule; D, Distal tubule; P, Proximal tubule; A, normal control (NC); B, obese control (OC); C, 200 mg *G. mangostana* flesh / kg body weight (GMF 200); D, 400 *G. mangostana* flesh / kg body weight (GMF 400); E, 600 *G. mangostana* flesh / kg body weight (GMF 600)

Histopathological investigation of kidney tissues after HFD feeding for 17 weeks resulted in dilation of glomerulus compared to NC rats (Fig. 2a and b). GMF supplemented rats at all doses showed normal structure of renal tissue (Fig. 2c, d and e).

## Discussion

The role of dietary fats in developing obesity is well established [24]. In this study, high-fat diet-induced obese rats were used as an experimental model for obesity. The experimental model produce obesity complications similar to human as obesity is developed from excess intake of calories which closely mimics the human diet. The high percentage of fat in the diet (40% in HFD compared to 30% in normal diet) provides excess calories intake [25] and resulted in the accumulation of body fat [7]. Consequently, HFD used in the present study successfully developed obesity in rats as evidenced from higher BMI compared to the rats fed with normal diet. Moreover, the HFD produced changes in metabolic parameters, as well as kidney and liver architectures. In the present study, the obese rats showed elevated level of plasma glucose and LDL-cholesterol level after 10 weeks of HFD induction. Similarly, Sprague-Dawley rats that were given 32% kcal of fat for 10 weeks showed significant increase in plasma LDL-cholesterol level as early as 3 weeks of induction period [26]. In addition, the obese rats in the present study developed dyslipidaemia which is characterized by the increased in triglyceride, total cholesterol and reduced HDL-cholesterol level after 17 weeks of HFD. This outcome is contrary with Poudyal et al. [27] and Ramli et al. [21] who found dyslipidaemia in high fat and high carbohydrate diet-induced obese rats after 8 weeks of induction. A possible explanation for this might be that the combination of excess carbohydrate and fat in the diet leads to higher rate of lipogenesis.

In obesity, the elevated levels of abdominal adipose tissues produces abundance of free fatty acid (FFA) released to the liver. These non-esterified fatty acids (NEFA) caused excessive production of triglyceride-rich lipoprotein (TG) particles like very low-density lipoprotein (VLDL). Consequently, liver, skeletal muscle and heart were exposed to the lipotoxic state and cause impairment to insulin-dependent metabolic process [28]. Increased VLDL is associated with increased synthesis of atherogenic LDL. High density lipoprotein cholesterol (HDL) can protect against development of atherosclerosis by reverse cholesterol transport from the vessel wall back to the liver and lessen inflammation by protecting LDL from being oxidized [29]. However, in obesity HDL decreased [30]. This is due to a reduction in lipolysis and hindered clearance of triglyceride-rich lipoprotein. Hence, this condition induces hyperinsulinemia, glucose intolerance and low plasma HDL levels [31, 32].

Besides, obesity leads to overproduction of reactive oxygen species from the increasing mechanical load and myocardial metabolism which eventually increased oxygen consumption. As a result, the balance between oxidant and antioxidant in the biological system is disrupted. The endogenous antioxidant defence system is reduced as the system is in favour of the oxidants [33]. As a result, the cell structure of lipids, proteins and DNA are damaged. In addition, the enlargement of adipocytes in obese people cause excess production of macrophage, a scavenging moribund adipocytes which is responsible for the production of inflammatory cytokines such as inducible nitric oxide synthase (iNOS) and interleukin-6 (IL-6) [34]. It was reported that the obese rats fed with high fat diet for 10 weeks had decreased their glutathione peroxidase (GPx) and superoxide dismutase (SOD) level [35]. Similarly, the current study found that obese rats had reduced plasma GPx and SOD as markers of antioxidant enzymes, as well as elevated level of plasma TNF-α and IL-6 as the pro-inflammatory markers. Therefore, our HFD-induced model showed metabolic and structural abnormalities that mimic human obesity.

Histopathological examination of liver and kidney in obese rats revealed comparable findings with previous research whereby dilation was observed in the hepatic central vein and branches of portal vein in rat fed with HFD (30%) for 8 to 12 weeks [36]. The finding suggests that HFD may act as major cause of liver steatosis. Additionally, results from the present study are in accordance with the previous study indicating that the kidney of rats that were given HFD for 3 months showed dilated blood vessels and Bowman’s capsule, mononuclear cell infiltration and degeneration in nephrons [37]. Human studies also demonstrated glomerular hypertrophy and glomerular capillary dilation in obese subjects [38]. It is suggested that the dilation of glomerulus after HFD is due to increase in metabolic demands in obese rats which consequently lead to glomerular hyperfiltration [39]. This finding broadly supports the work of other studies in this area linking obesity and glomerular size [40, 41] and adiposity and glomerular hyperfiltration [42].

With the exception of increased level of plasma glucose, GMF supplementation attenuated metabolic abnormalities and structural changes due to high fat feeding. GMF-supplemented rats reduced body weight and inflammation, improved lipid profile with no changes in HDL-cholesterol, enhanced antioxidant defence system and decreased hepatic fat accumulation and ameliorated renal abnormalities. Nevertheless, the most notable result from our study is the reduction of body weight and food intake while improving some signs of metabolic syndrome. GMF supplementation decreased food intake and total energy intake causing a reduction in body weight and other cardio-metabolic risk factors. We observed that GMF supplemented groups showed the lowest weight gain compared to control rats between week 17 and week 10. Similar results had been reported by Azman et al. [35] whereby obese rats supplemented with *Tamarindus indica* pulp at 25 mg/kg and 50 mg/kg showed a reduction in their body weight. The report also revealed that total food intake of the animals did not relate to total dietary caloric consumed. Our data provides us with the fact that supplementation of GMF in obese rats reduced the amount of food and total dietary calorie consumed and subsequently suppressed the excessive body weight gain due to HFD. This observations support the hypothesis that *G. mangostana* possesses weight reducing properties.

Nevertheless, Benton and Young [43] reported that the decreasing in body weight due to reduction in energy intake is a short term effect. Energy balance is a complex system and involves the interactions of many factors other than energy intake and energy expenditure [44]. Therefore, it is important to emphasize the physiological and psychological factors including satiating effect which may influence the amount of foods consumed. Among the contribution factors are cognitive, sensory and physiological signals during consumption and the macronutrients components of foods [45]. GMF contain 5.1% fiber in the flesh [46, 47]. The high level of fiber in GMF might contribute to satiety effects. Dietary fiber especially soluble fiber that present in significant amount in fruit have been shown to improve post-prandial satiety and reduce subsequent hunger in short term studies [48]. In fact, previous research showed the potential role of whole fruit in reducing obesity symptoms from its relatively low level of energy density and high content of dietary fiber [49]. It can thus be suggested that the mechanism of body weight reduction is due to the metabolic effects of fiber which increase the nutrient breakdown and release from the foods and contribute to satiating effect [50] as shown in our previous study [51].

In addition to the metabolic effects elicited by dietary fiber, the bioactive compounds in *G. mangostana* such as xanthones, anthocyanin and phenolic acids [52] may play a pivotal role in normalizing the metabolic abnormalities in obese rats. Available evidences suggested that the polyphenols from mangoesteen extract including its xanthones exerted good pharmacological effects in animal model of metabolic syndromes, diabetes and obesity [53, 54]. The most abundant xanthones in the pericarp of mangosteen is α-mangostin which has been shown to possess strong cardioprotective, antioxidant, anti-inflammatory and anti-obesity agents [55]. These beneficial effects are clearly seen in the present study whereby the antioxidant enzymes and total antioxidant capacity (TAC) increases while total cholesterol, LDL-cholesterol and triglyceride level in obese rats decreases following GMF supplementation. Our results are consistent with those of Choi et al. [56] and Chae et al. [57] who found a reduction of free fatty acids (FFA), total cholesterol and LDL-cholesterol in obese mouse supplemented with 50 mg/kg α-mangostin or 200 mg/kg peel extract. Previous study has shown that the supplementation of α-mangostin in obese mice reduced their body weight through the regulation of SirT-1-AMPK (Sirtuin 1–5’AMP-activated protein kinase) and PPARγ (peroxisome proliferator-activated receptor-γ) pathways [56]. Based on the studies, it is proposed that the antiobesity effect of GMF is associated with the increase in lipid metabolism that lead to the reduction in body fat mass and other related metabolic complications. It is possible; therefore, the combined effect of α-mangostin and fiber may be responsible for the GMF-induced reduction of body weight and the improvement of lipid profile in the present study. Thus, the limitation of the present study is the lack of capacity to determine the effect of specific compounds in GMF that mediate the observed positive results.

In the present study, GMF was observed to enhance endogenous antioxidant system as evidenced from the elevated level of antioxidant enzymes. These effects suggest the ability of GMF to scavenge the free radicals generated from HFD and illustrate the efficiency of GMF in the deactivation of hydrogen peroxide in biological system. In addition, the reductions of TNF-α and IL-6 levels after GMF supplementation reflect inflammation decreased in obese rats. A comparable finding was reported by Bumrungpert et al. [18] whereby human adipocytes treated with α-mangostin showed a decreased in the expression of inflammatory genes. Besides, daily consumption of mangosteen-based drink for 30 days in healthy adult demonstrated 46% reduction of inflammatory markers [58]. A part from that, the treatment with 800 mg/kg ethanolic extract of mangosteen pericarp induced protection from atherosclerosis in hypercholestromic rats by maintaining the expression of eNOS (endothelial nitric oxide synthase) and reducing hydrogen peroxide and HIF-1α (hypoxia-inducible factor 1-alpha) that eventually leads to reduce vasa vasorum angiogenesis [13, 59]. On the contrary, α-mangostin was unable to scavenge hydrogen peroxide; instead, the compound ameliorated 3-nitropropionic acid-induced reactive oxygen species formation and provides renoprotective effects against cisplatin (cDDP; cis-diamminedichloroplatinum II)-induced nephrotoxicity in rats [60]. Nevertheless, the inhibition of lipid peroxidation and enhancement of antioxidant defence system shown in injury-induced myocardial infarction in rats following α-mangostin pre-treatment could reflect the potent antioxidant and anti-inflammatory action of this xanthones [61]. It can thus be proposed that GMF has the potential to attenuate abnormalities caused by high fat diet-induced obesity in rats based on the positive results observed from the present study.

## Conclusion

The results of this study demonstrate that supplementation of GMF in high fat diet-induced obese rats reduced body weight and attenuated the metabolic and structural changes in liver and kidney of obese rats. Therefore, GMF may have potential to ameliorate the complications related to high fat diet-induced obesity in rats. However, the results warrant further research on pharmacokinetics and pharmacodynamic, as well as the underlying mechanism using molecular approach, especially on the effects of specific bioactive compounds in GMF on obesity. Furthermore, it is also crucial to determine the effect GMF on pancreatic lipase activity as the inhibition of its activity may reduce fat absorption, and possess beneficial effects on obesity.

## Data Availability

The datasets used and/or analysed during the current study available from the corresponding author on reasonable request.

## Abbreviations

ANOVA: Analysis of Variance
BMI: body mass index
CDDP: cisplatin
ELISA: enzyme-linked immunosorbent assays
eNOS: endothelial nitric oxide synthase
FFA: free fatty acid
GMF: *Garcinia mangostana*
GPx: glutathione peroxidase
HDL: high density lipoprotein
HF: high fat diet
HIF-1α: hypoxia-inducible factor 1-alpha
IL-6: inteleukin-6
LDL: low density lipoprotein
LSD: Least Significant Difference
NC: normal control group
NEFA: non-esterified fatty acids
OC: obese control group
PPARγ: peroxisome proliferator-activated receptor-γ
SirT1-AMPK: Sirtuin 1–5’AMP-activated protein kinase
SOD: superoxide dismutase
TAC: total antioxidant capacity
TG: triglyceride
TNF-α: alpha-tumor necrosis
VLDL: very low-density lipoprotein
